# Reduced Dimension Based Two-Dimensional DOA Estimation with Full DOFs for Generalized Co-Prime Planar Arrays

**DOI:** 10.3390/s18061725

**Published:** 2018-05-27

**Authors:** Fenggang Sun, Peng Lan, Guowei Zhang

**Affiliations:** 1 College of Information Science and Engineering, Shandong Agricultural University, Tai’an 271018, China; 2 Information School, Shandong University of Political Science and Law, Jinan 250014, China; zhanggw_xinyuan@sdupsl.edu.cn

**Keywords:** two-dimensional, DOA estimation, generalized co-prime planar array, partial spectral search, iterative approach, degrees-of-freedom (DOFs)

## Abstract

In this paper, we investigate the problem of two-dimensional (2D) direction-of-arrival (DOA) estimation for generalized co-prime planar arrays. The classic multiple signal classification (MUSIC)-based methods can provide a superior estimation performance, but suffer from a tremendous computational burden caused by the 2D spectral search. To this end, we reduce the 2D problem into a one-dimensional (1D) one and propose a reduced dimension partial spectral search estimation method, which can compress the search region into a small 1D sector. Moreover, the proposed method can utilize the full information of the entire array without degrees-of-freedom loss. Furthermore, an iterative approach is also proposed to reduce complexity and improve performance. Simulation results show that the proposed methods can provide improved performance with substantially reduced complexity, as compared to other state-of-the-art methods.

## 1. Introduction

Two-dimensional (2D) direction-of-arrival (DOA) estimation has played an important role in the area of array signal processing [1,2,3]. Various methods have been used in radar, sonar and other applications, such as multiple signal classification (MUSIC) [4], quaternion-MUSIC [5] and the estimation of signal parameters via rotational invariance technique (ESPRIT) [6]. Among these methods, MUSIC is regarded as one of the most representative techniques due to its high resolution and flexibility for arbitrary arrays. However, the MUSIC approach usually suffers from a tremendous computational burden due to the 2D spectral search [7].

For 2D DOA estimation, uniform array geometries are commonly used, e.g., L-shaped array [8], rectangular array [9] and parallel linear array [10]. Recently, the co-prime array [11,12,13], consisting of two co-prime subarrays, has drawn much attention due to its extended aperture and improved resolution [14,15,16]. To estimate 2D DOAs, the co-prime planar array geometry was designed in [17], which was then extended to a generalized form in [18]. According to the relation between the true and their ambiguous angles, a partial spectral search (2D PSS) method was proposed in [17,18], which can compress the search region into a small 2D sector to reduce complexity. Since the 2D spectral search is still required, the complexity of the PSS method is still high. To address this issue, a reduced dimensional-based PSS method (RD-PSS) was proposed in [19], where only a one-dimensional partial spectral search is performed. For co-prime planar arrays, a polynomial root finding-based method was proposed in [20], which can avoid the spectral search step. Then, the co-prime array concept was extended to multiple input multiple output radars to estimate 2D DOAs with enhanced degrees-of-freedom (DOFs) [21].

However, the methods in [17,18,19,20] require that each subarray estimates the DOAs individually by their own data and obtains the estimate by combining the results of the two subarrays. For the individual estimate of each subarray, the mutual information of the received data between the two subarrays is lost; therefore, the estimation accuracy is degraded, and the DOFs are sacrificed.

In this paper, we present a computationally-efficient reduced dimension-based DOA estimation method for generalized co-prime planar arrays. Different from the separate processing for each subarray [17,18,19,20], we use the received data of the two subarrays as a whole, which can avoid mutual information loss. In terms of the achievable DOFs, the methods in [17,18,19,20] only utilize their own data for each subarray, and the DOFs will be halved. By contrast, we jointly exploit the entire data, where the full DOFs can be achieved. To deal with the computation burden, we reduce the 2D spectral search into a 1D one and then propose a 1D PSS-based DOA estimation, where the search region is compressed into a small sector. Furthermore, we also propose an iterative approach to reduce complexity and improve accuracy. Simulations have shown that the proposed method can provide superior estimation performance with substantially reduced complexity, as compared with other state-of-the-art methods.

## 2. System Model

**Figure 1:**
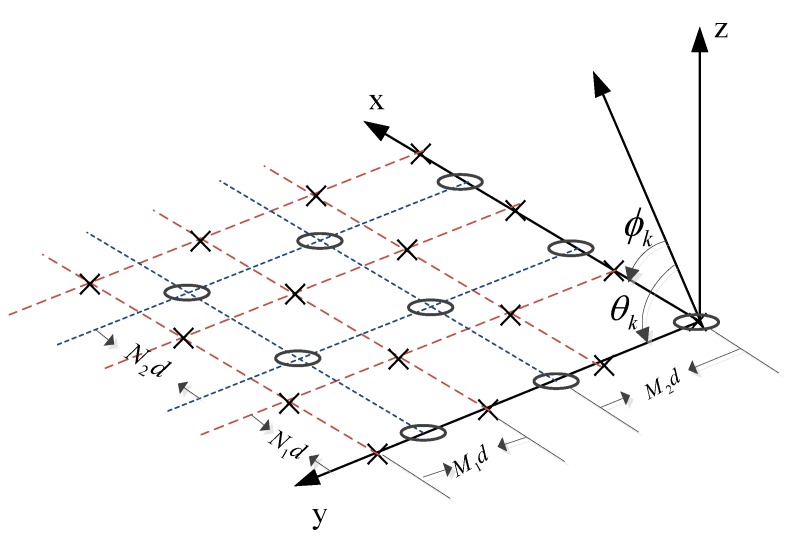
System model of the considered generalized coprime planar array, where $d=\frac{\lambda }{2}$.

In this paper, we consider a generalized coprime planar array, as shown in Figure 1, which consists of two uniform rectangular subarrays. The *i*-th (i=1,2) subarray contains $N_{i}\times M_{i}$ sensors, where $N_{i}$ and $M_{i}$ are the numbers of sensors in the *x*-axis and *y*-axis direction, respectively. The corresponding inter-element spacings are dxi=Ni˜λλ22 and dyi=Mi˜λλ22, where $i+\tilde{i}=3$, $N_{1}$ and $N_{2}$, $M_{1}$ and $M_{2}$ are coprime integers and λ is the wavelength. Since the two subarrays only overlap at the origin position, the total number of sensors is $M=N_{1}M_{1}+N_{2}M_{2}-1$.

Suppose that *K* far-field narrowband signals impinge on the array from directions $(\theta_{k},\phi_{k}),k=1,2,\cdots ,K$, where $\theta_{k}$ denotes the angle between the incident direction and the *y*-axis and $\phi_{k}$ is the angle between the incident direction and the *x*-axis. The received signal of the *i*-th subarray at the *t*-th snapshot (t=1,2,⋯,N) can be given as:

(1) $$x_{i}\left(t\right)=\sum_{k=1}^{K}a_{i}\left(\theta_{k},\phi_{k}\right)s_{k}\left(t\right)+n_{i}\left(t\right)=A_{i}s\left(t\right)+n_{i}\left(t\right),$$

where $s\left(t\right)={\left[s_{1}\left(t\right),s_{2}\left(t\right),\cdots ,s_{K}\left(t\right)\right]}^{T}$ is the signal vector with $s_{k}\left(t\right)$ being the signal of the *k*-th source, which follows the Gaussian distribution with zero mean and unit variance. $n_{1}\left(t\right)$ and $n_{2}\left(t\right)$ are the additive white Gaussian noise (AWGN) vectors with zero mean and variance $\sigma_{n}^{2}$ and are assumed to be uncorrelated with the incident signals $s\left(t\right)$. $A_{i}=\left[a_{i}\left(\theta_{1},\phi_{1}\right),a_{i}\left(\theta_{2},\phi_{2}\right),\cdots ,a_{i}\left(\theta_{K},\phi_{K}\right)\right]\in C^{N_{i}M_{i}\times K}$ denotes the manifold matrix of the *i*-th subarray and $a_{i}\left(\theta_{k},\phi_{k}\right)=a_{y_{i}}\left(\theta_{k}\right)⊗a_{x_{i}}\left(\phi_{k}\right)$, where $a_{x_{i}}\left(\phi_{k}\right)$ and $a_{y_{i}}\left(\theta_{k}\right)$ are the steering vectors of the *i*-th (i=1,2) subarray along the *x*-axis and *y*-axis [18], respectively, and can be represented as:

(2) $$a_{x_{i}}\left(\phi_{k}\right)={\left[1,e^{-j\pi N_{\tilde{i}}\cos\phi_{k}},\ldots ,e^{-j\pi \left(N_{i}-1\right)N_{\tilde{i}}\cos\phi_{k}}\right]}^{T},$$

(3) $$a_{y_{i}}\left(\theta_{k}\right)={\left[1,e^{-j\pi M_{\tilde{i}}\cos\theta_{k}},\ldots ,e^{-j\pi \left(M_{i}-1\right)M_{\tilde{i}}\cos\theta_{k}}\right]}^{T},$$

with $i+\tilde{i}=3$ and ${\left(\cdot \right)}^{T}$ being the transpose operation. Concatenating the array measurements $x_{1}\left(t\right)$ and $x_{2}\left(t\right)$, we can get:

(4) $$\begin{array}{cc} x(t) & =\left[\begin{array}{c} x_{1}\left(t\right)\\ x_{2}\left(t\right) \end{array}\right]=\left[\begin{array}{c} A_{1}\\ A_{2} \end{array}\right]s\left(t\right)+\left[\begin{array}{c} n_{1}\left(t\right)\\ n_{2}\left(t\right) \end{array}\right]\\ & =\mathrm{As}\left(t\right)+n\left(t\right), \end{array}$$

where $A={\left[A_{1}^{T},A_{2}^{T}\right]}^{T}$ and $n\left(t\right)={\left[n_{1}^{T}\left(t\right),n_{2}^{T}\left(t\right)\right]}^{T}$. The covariance matrix can be given by:

(5) $$R=E\left[x\left(t\right)x^{H}\left(t\right)\right]=AR_{s}A^{H}+\sigma_{n}^{2}I_{M+1},$$

where $R_{s}=E\left[s\left(t\right)s^{H}\left(t\right)\right]=diag\left(\sigma_{1}^{2},\sigma_{2}^{2},\cdots ,\sigma_{K}^{2}\right)$ is the source covariance matrix. ${\left(\cdot \right)}^{H}$ denotes conjugate transpose and $I_{M+1}$ is a (M+1)×(M+1) identity matrix.

2D MUSIC is applicable to estimate the DOAs for the entire array [17], and the eigen-value decomposition (EVD) of R is:

(6) $$R=U_{s}\Sigma_{s}U_{s}^{H}+U_{n}\Sigma_{n}U_{n}^{H},$$

where the subscripts *s* and *n* denote the signal- and noise-subspace, respectively, and $\Sigma_{s}$ and $\Sigma_{n}$ are two diagonal matrices containing the significant and zero eigenvalues, respectively. The DOAs can be estimated by:

(7) $$\min_{\theta ,\phi }f\left(\theta ,\phi \right)=a^{H}\left(\theta ,\phi \right)U_{n}^{}U_{n}^{H}a\left(\theta ,\phi \right),$$

where $a\left(\theta ,\phi \right)={\left[a_{1}^{T}\left(\theta ,\phi \right),a_{2}^{T}\left(\theta ,\phi \right)\right]}^{T}$ is the steering vector of the entire array.

## 3. Proposed Algorithms

In this section, we firstly propose a reduced dimensional approach to estimate 2D DOAs by utilizing the full information without DOF loss and then propose an iterative approach to further reduce the computational complexity and improve the estimation performance.

### 3.1. Full Information-Based Reduced Dimension Partial Spectral Search Approach

As the manifold matrix and signal subspace can span the same space, we have:

(8) $$U_{s}=\left[\begin{array}{c} A_{1}\\ A_{2} \end{array}\right]T=\left[\begin{array}{c} U_{s1}\\ U_{s2} \end{array}\right],$$

where T is a non-singular matrix. By defining the transformation matrices as $H_{1}=U_{s2}U_{s1}^{+}=A_{2}TT^{-1}A_{1}^{+}=A_{2}A_{1}^{+}$ and $H_{2}=U_{s1}U_{s2}^{+}=A_{1}TT^{-1}A_{2}^{+}=A_{1}A_{2}^{+}$, the relation between $A_{1}$ and $A_{2}$ can be given as [22]:

(9) $$A_{2}=H_{1}A_{1},\;A_{1}=H_{2}A_{2},$$

where ${\left(\right)}^{+}$ represent the pseudo-inverse operation. The MUSIC spectrum f(θ,ϕ) is then equivalent as:

(10) $$\begin{array}{cc} f(\theta ,\phi ) & ={\left[\begin{array}{c} a_{1}\left(\theta ,\phi \right)\\ H_{1}a_{1}\left(\theta ,\phi \right) \end{array}\right]}^{H}U_{n}^{}U_{n}^{H}\left[\begin{array}{c} a_{1}\left(\theta ,\phi \right)\\ H_{1}a_{1}\left(\theta ,\phi \right) \end{array}\right]\\ & ={\left[\begin{array}{c} H_{2}a_{2}\left(\theta ,\phi \right)\\ a_{2}\left(\theta ,\phi \right) \end{array}\right]}^{H}U_{n}^{}U_{n}^{H}\left[\begin{array}{c} H_{2}a_{2}\left(\theta ,\phi \right)\\ a_{2}\left(\theta ,\phi \right) \end{array}\right]. \end{array}$$

As $a_{i}\left(\theta ,\phi \right)=a_{yi}\left(\theta \right)⊗a_{xi}\left(\phi \right)=\left(a_{yi}\left(\theta \right)⊗I_{N_{i}}\right)a_{xi}\left(\phi \right)$ holds [23], with ⊗ denoting the Kronecker product, we have:

(11) $$\begin{array}{c} a_{1}\left(\theta ,\phi \right)=\left(a_{y1}\left(\theta \right)⊗I_{N_{1}}\right)a_{x1}\left(\phi \right), \end{array}$$

(12) $$\begin{array}{cc} a_{2}\left(\theta ,\phi \right) & =a_{y2}\left(\theta \right)⊗a_{x2}\left(\phi \right)=R\left(a_{x2}\left(\phi \right)⊗a_{y2}\left(\theta \right)\right)\\ & =R\left(a_{x2}\left(\phi \right)⊗I_{M_{2}}\right)a_{y2}\left(\theta \right), \end{array}$$

where R is an exchange matrix and is defined as:

$$R=\left[\begin{array}{c} I_{N_{2}}⊗e_{1,M_{2}}^{T}\\ I_{N_{2}}⊗e_{2,M_{2}}^{T}\\ \;\;\vdots \\ I_{N_{2}}⊗e_{M_{2},M_{2}}^{T} \end{array}\right].$$

By inserting Equations (11) and (12) into (10), we have:

(13) $$\begin{array}{cc} f_{}\left(\theta ,\phi \right) & =a_{x1}^{H}\left(\phi \right)P\left(\theta \right)a_{x1}\left(\phi \right)\\ & =a_{y2}^{H}\left(\theta \right)Q\left(\phi \right)a_{y2}\left(\theta \right), \end{array}$$

where

$$K\left(\theta \right)=a_{y1}\left(\theta \right)⊗I_{N_{1}},$$

$$L\left(\phi \right)=a_{x2}\left(\phi \right)⊗I_{M_{2}},$$

$$P\left(\theta \right)={\left[\begin{array}{c} K\left(\theta \right)\\ H_{1}K\left(\theta \right) \end{array}\right]}^{H}U_{n}^{}U_{n}^{H}\left[\begin{array}{c} K\left(\theta \right)\\ H_{1}K\left(\theta \right) \end{array}\right],$$

$$Q\left(\phi^{}\right)={\left[\begin{array}{c} H_{2}\mathrm{RL}\left(\phi \right)\\ \mathrm{RL}\left(\phi \right) \end{array}\right]}^{H}U_{n}^{}U_{n}^{H}\left[\begin{array}{c} H_{2}\mathrm{RL}\left(\phi \right)\\ \mathrm{RL}\left(\phi \right) \end{array}\right].$$

To minimize $f\left(\theta ,\phi \right)=a_{x1}^{H}\left(\phi \right)P\left(\theta \right)a_{x1}\left(\phi \right)$, we need to eliminate the trivial solution $a_{x1}\left(\theta \right)=0$ by constraining $e_{1,N_{1}}^{H}a_{x1}\left(\phi \right)=1$, where $e_{1,M}$ denotes a M×1 all-zero vector, except for the first term being one. Thus, we have,

(14) $$\begin{array}{c} \begin{array}{c} \begin{array}{cc} \min & a_{x1}^{H}\left(\phi \right)P\left(\theta^{}\right)a_{x1}\left(\phi \right) \end{array}\\ \begin{array}{cc} s.t. & e_{1,N_{1}}^{H}a_{x1}\left(\phi \right)=1. \end{array} \end{array} \end{array}$$

The optimal solution can be given as:

(15) $$\begin{array}{c} a_{x1}\left(\phi^{}\right)=\frac{P^{-1}\left(\theta^{}\right)e_{1,N_{1}}^{}}{e_{1,N_{1}}^{H}P^{-1}\left(\theta^{}\right)e_{1,N_{1}}^{}}, \end{array}$$

and then, f(θ,ϕ) is simplified as:

(16) $$f\left(\theta ,\phi \right)=\frac{1}{e_{1,N_{1}}^{H}P^{-1}\left(\theta \right)e_{1,N_{1}}^{}}.$$

Equivalently, to minimize $f\left(\theta ,\phi \right)=a_{y2}^{H}\left(\theta \right)Q\left(\phi^{}\right)a_{y2}\left(\theta \right)$, we can formulate the following optimization problem,

(17) $$\begin{array}{c} \begin{array}{c} \begin{array}{cc} \min_{\theta } & a_{y2}^{H}\left(\theta \right)Q\left(\phi^{}\right)a_{y2}\left(\theta \right) \end{array}\\ \begin{array}{cc} s.t. & e_{1,M_{2}}^{H}a_{y2}\left(\theta \right)=1, \end{array} \end{array} \end{array}$$

and the best solution is:

(18) $$\begin{array}{c} a_{y2}\left(\theta^{}\right)=\frac{Q^{-1}\left(\phi^{}\right)e_{1,M_{2}}^{}}{e_{1,M_{2}}^{H}Q^{-1}\left(\phi^{}\right)e_{1,M_{2}}^{}}. \end{array}$$

Then, f(θ,ϕ) can be modified as:

(19) $$f\left(\theta ,\phi \right)=\frac{1}{e_{1,M_{2}}^{H}Q^{-1}\left(\phi \right)e_{1,M_{2}}^{}}.$$

From the one-dimension spectral search of (16) (or (19)), we can get the estimate of θ (or ϕ). Then, by inserting these estimates into (15) (or (18)), we can obtain the corresponding estimate of ϕ (or θ) from the phases [19]. Therefore, the angles are paired automatically.

Due to the inter-element spacing being larger than half a wavelength, there exist multiple ambiguous angles for each true DOA. Specifically, the relations between the true and ambiguous angles are given as [17]:

(20) $$\begin{array}{c} \cos\theta_{k}-\cos\theta_{k,i}^{a}=\frac{2k_{i}}{M_{\tilde{i}}},\;\cos\phi_{k}-\cos\phi_{k,i}^{a}=\frac{2l_{i}}{N_{\tilde{i}}}, \end{array}$$

where $\left(\theta_{k,i}^{a},\phi_{k,i}^{a}\right)$ denotes the ambiguous angle with respect to the true DOA $\left(\theta_{k},\phi_{k}\right)$ for the *i*-th subarray and $k_{i}$ and $l_{i}$ are integers. From the relation (20), the one-dimension spectral search can be limited to a small sector. To estimate θ in (16) (or ϕ in (19)), we just need to search over an arbitrary 22M2M2 interval (or 22N1N1) in the cosine domain. Meanwhile, the others can be calculated by (20). Finally, according to the co-prime property, we can obtain the true DOAs without ambiguity [17]. As the proposed method can fully utilize the information of the entire array, we refer to it as the full information-based reduced dimension partial spectral search approach (FuRD-PSS).

### 3.2. Iterative Approach

The FuRD-PSS approach can limit the search region to a small one-dimensional sector, which can greatly reduce the complexity. To further reduce the complexity, a two-step iterative approach is proposed here.

Firstly, we obtain a coarse estimate from the FuRD-PSS approach as $\left(\theta^{\left(0\right)},\phi^{\left(0\right)}\right)$. Then, for the *i*-th iteration,

Step 1: Fix $\phi^{\left(i-1\right)}$, and update θ.

The derivation of $f(\theta ,\phi^{\left(i-1\right)})$ with respect to θ is $\frac{\partial f}{\partial \theta }=\frac{\partial {\left[a_{y2}\left(\theta \right)\right]}^{T}}{\partial \theta }Q^{T}\left(\phi^{\left(i-1\right)}\right)a_{y2}^{*}\left(\theta \right)$. Then, θ can be updated as:

(21) $$\theta^{\left(i\right)}=\theta^{\left(i-1\right)}-\Delta_{\theta }\frac{\partial f}{\partial \theta }\left|{}_{\theta =\theta^{\left(i-1\right)}}\right.,$$

where $\Delta_{\theta }$ is the update step for θ.

Step 2: With the estimated $\theta^{\left(i\right)}$, update ϕ.

The derivation of $f(\theta^{\left(i\right)},\phi )$ with respect to $\frac{\partial f}{\partial \phi }=\frac{\partial {\left[a_{x1}\left(\phi \right)\right]}^{T}}{\partial \phi }P^{T}\left(\theta^{\left(i\right)}\right)a_{x1}^{*}\left(\phi \right)$. Then ϕ can be updated as:

(22) $$\phi^{\left(i\right)}=\phi^{\left(i-1\right)}-\Delta_{\phi }\frac{\partial f}{\partial \phi }\left|{}_{\phi =\phi^{\left(i-1\right)}}\right.,$$

where $\Delta_{\phi }$ is the update step for ϕ.

As f(θ,ϕ) is quadric with respect to $a_{x_{1}}\left(\phi \right)$ and $a_{y_{2}}\left(\theta \right)$, the iterative approach converges within a few iterations, which can improve the estimation accuracy efficiently. It is noted that the iterative approach requires a coarse estimate of the true DOAs a priori, which can reduce the complexity. Then, the finer estimation can be obtained through limited iterations.

### 3.3. Procedure of the Proposed Algorithms

To sum up, the proposed FuRD-PSS method is shown as follows:

1. Estimate the covariance matrix R as (5).
2. Perform EVD of R as (6) and select the signal subspace with respect to the *K* largest eigenvalues.
3. Construct the MUSIC spectrum f(θ,ϕ) (10) with respect to $a_{1}$ or $a_{2}$, according to the relations between $A_{1}$ and $A_{2}$ (10).
4. Obtain the estimate of θ in (16) and ϕ in (19) through 1D partial spectral search.
5. Calculate the estimate of ϕ and θ according to (15) and (18).
6. Recover all the ambiguous angles with respect to θ and ϕ, according to (20).
7. Combine the results of the two subarrays to obtain the final estimate, according to the co-primeness of the two subarrays.

Then, to reduce the complexity, the iterative approach is further performed based on the coarse estimate of FuRD-PSS. Specifically,

8. Obtain a coarse estimate from the FuRD-PSS approach as $\left(\theta^{\left(0\right)},\phi^{\left(0\right)}\right)$.
9. Fix $\phi^{\left(i-1\right)}$, and obtain the derivation of $f(\theta ,\phi^{\left(i-1\right)})$ with respect to θ, then update θ as (21).
10. With the estimated $\theta^{\left(i\right)}$, update ϕ according to (22).

## 4. Complexity Analysis

We now compare the computational complexity of the proposed methods with other existing methods.

The complexities of the MUSIC-based methods are mainly caused by EVD and spectral search, where the complexity for EVD is $O\left(M^{3}\right)$ [24]. For 2D MUSIC, the 2D spectral search is required, where the complexity is $O\left(J_{\theta }J_{\phi }M\left(M-K\right)\right)$ [17], and $J_{\theta }$ and $J_{\phi }$ are numbers of the searching grids for θ and ϕ. Therefore, the total complexity can be given as:

$$C_{2DMUSIC}=O\left(M^{3}+J_{\theta }J_{\phi }M\left(M-K\right)\right).$$

For the FuRD-PSS method, the complexities for estimating θ (16) and ϕ (19) through 1D partial search are $O\left(\frac{J_{\theta }}{M_{2}}N_{1}M\left(M-K\right)\right)$ and $O\left(\frac{J_{\phi }}{N_{1}}M_{2}M\left(M-K\right)\right)$, respectively. The total complexity is given as:

$$C_{FuRD-PSS}=O\left(M^{3}+\frac{J_{\theta }}{M_{2}}N_{1}M\left(M-K\right)+\frac{J_{\phi }}{N_{1}}M_{2}M\left(M-K\right)\right).$$

For the iterative approach, a coarse search step is required, and the complexity is given as $O\left(M^{3}+\left(\frac{J_{\theta }^{c}}{M_{2}}N_{1}+\frac{J_{\phi }^{c}}{N_{1}}M_{2}\right)M\left(M-K\right)\right)$, where $J_{\theta }^{c}$ ($\ll J_{\theta }$) and $J_{\phi }^{c}$ ($\ll J_{\phi }$) are the searching numbers. The following iterative process of (21) and (22) requires the complexity $O\left(M_{2}M\left(M-K\right)\right)$ and $O\left(N_{1}M\left(M-K\right)\right)$, respectively. Therefore, the total complexity for the iterative approach is given as:

$$C_{iterative}=O\left(M^{3}+\left(\frac{J_{\theta }^{c}}{M_{2}}N_{1}+\frac{J_{\phi }^{c}}{N_{1}}M_{2}+J_{I}\left(M_{2}+N_{1}\right)\right)M\left(M-K\right)\right),$$

where $J_{I}$ denotes the number of iterations.

For clarity, we give the complexities of different methods in Table 1. It is noted that the proposed FuRD-PSS and the iterative approach reduce the 2D search into a small 1D sector, which can substantially reduce the complexity.

## 5. Simulation Results

In this section, we compare the performance of the proposed methods with other methods, including 2D MUSIC for a uniform rectangle array, RD-PSS [19] and the stochastic CRB [18]. The searching grids for θ and ϕ are both set as $0.1^{\circ }$, and the coarse grids for the iterative approach are both set as $0.5^{\circ }$. The number of iterations for the iterative approach is $J_{I}=10$. We use the root mean square error (RMSE) to measure the estimation performance, defined as:

(23) $$RMSE=\sqrt{\frac{1}{SK}\sum_{s=1}^{S}\sum_{k=1}^{K}\left(\left(\theta_{k}-{\hat{\theta }}_{k}^{s}\right)+\left(\phi_{k}-{\hat{\phi }}_{k}^{s}\right)\right)},$$

where *S* denote the times of Monte Carlo trials, and we set S=200 in the following simulations. $\left({\hat{\theta }}_{k}^{s},{\hat{\phi }}_{k}^{s}\right)$ denote the estimate of the *k*-th source $\left(\theta_{k},\phi_{k}\right)$ of the *s*-th trial.

**Figure 2:**
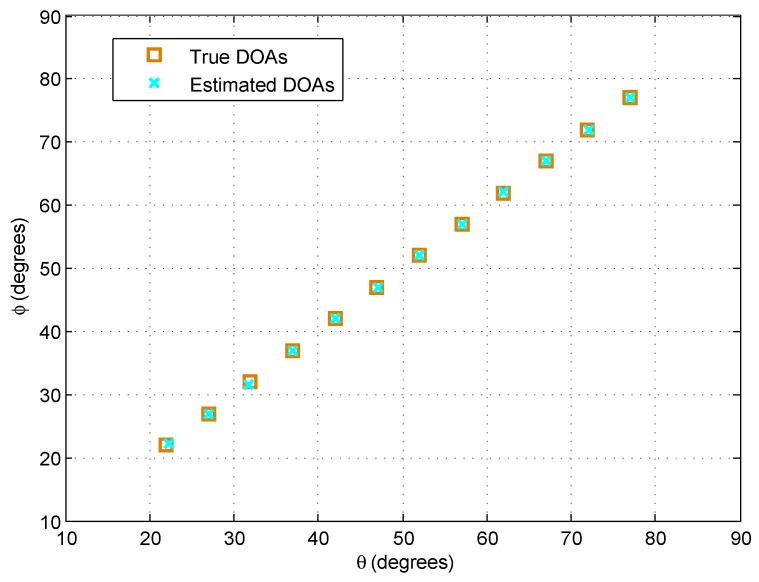
DOA estimation results for the proposed algorithm with K=12 sources and $M_{1}=N_{1}=3,$$M_{2}=N_{2}=7$.

Figure 2 illustrates the detection performance of the proposed algorithm, where K=12 sources are uniformly distributed between $\left(20^{\circ },20^{\circ }\right)$ and $\left(77^{\circ },77^{\circ }\right)$. The snapshot number is N=200, and the signal-to-noise ratio (SNR) is 10 dB. As can be observed, the proposed method can detect more than $O\left(\min(M_{1}N_{1},M_{2}N_{2})\right)$ sources, while 2D-PSS [17] and RD-PSS fail to detect any in this case. This is because the proposed algorithm can utilize the full information of the entire array and provide $O\left((M_{1}N_{1}+M_{2}N_{2})\right)$ DOFs. Meanwhile, 2D-PSS and RD-PSS only provide $O\left(\min(M_{1}N_{1},M_{2}N_{2})\right)$ DOFs, which are limited by the sensor number with the less sensors.

**Figure 3:**
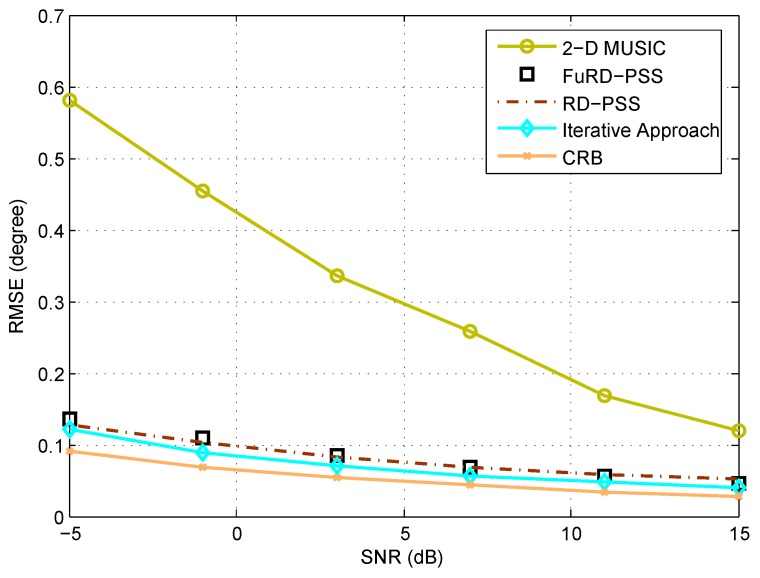
RMSE comparison of different methods versus SNR.

**Figure 4:**
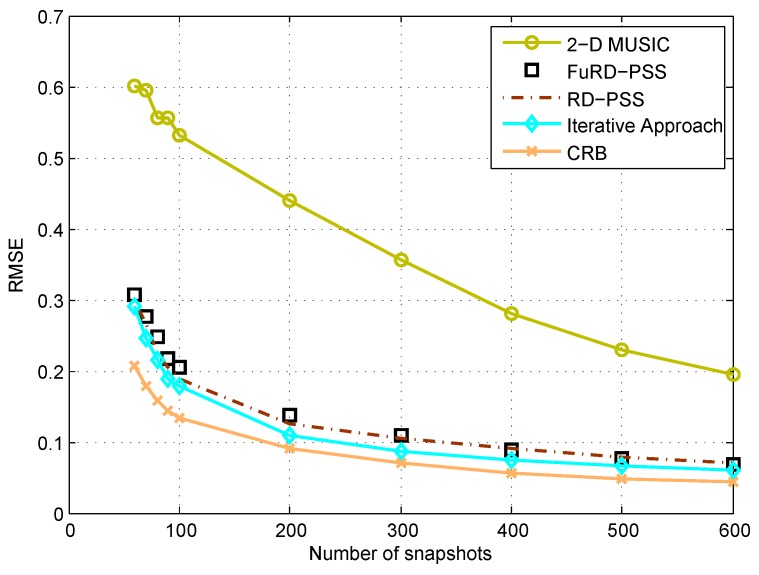
RMSE comparison of different methods versus the number of snapshots.

Figure 3 and Figure 4 show the root mean square error (RMSE) performance of different methods with respect to the SNR and the number of snapshots, respectively. Here, we consider a generalized coprime planar array, with $M_{1}=4$, $N_{1}=4$, $M_{2}=5$, $N_{2}=3$ and K=5 sources with the DOAs uniformly distributed within the range $\left(20.3^{\circ },25.3^{\circ }\right)$ and $\left(48.3^{\circ },57.3^{\circ }\right)$, which are kept the same during each Monte Carlo trial. The snapshot number is set as N=200 for Figure 3, and the SNR is 0 dB for Figure 4. As can be observed, the RMSE performance is improved with the increase of SNR and snapshot number. Specifically, FuRD-PSS is much better than 2D MUSIC and provides almost the same performance as RD-PSS. When the iterative approach is performed, the performance can be further improved. In terms of the complexity, the complexities for 2D MUSIC, RD-PSS, FuRD-PSS and the iterative approach are given as $O\left(2.43\times 10^{9}\right)$, $O\left(4.63\times 10^{5}\right)$, $O\left(2.79\times 10^{6}\right)$ and $O\left(6.48\times 10^{5}\right)$, respectively. Therefore, the complexities of FuRD-PSS and the iterative approach are much lower than that of 2D MUSIC. As compared to RD-PSS, the complexities become slightly greater. However, as shown in Figure 2, the proposed methods can provide higher DOFs.

## 6. Conclusions

In this paper, we have addressed the 2D DOA estimation issue for generalized co-prime planar arrays. By considering the full information of the entire array, we have proposed reduced dimensional-based estimation methods with improved performance and no degrees-of-freedom loss. Specifically, we reduce the 2D problem into a 1D one and propose a 1D partial spectral search estimation method to reduce complexity. With a coarse estimate, an iterative approach is further proposed to improve estimation performance. Simulation results are illustrated to show the superiority of the proposed methods.

## Figures and Tables

**Table 1:** The complexities of different methods. FuRD-PSS, full information-based reduced dimension partial spectral search.

Methods	Complexity
MUSIC	$O\left(M^{3}+J_{\theta }J_{\phi }M\left(M-K\right)\right)$
FuRD-PSS	$O\left(M^{3}+\frac{J_{\theta }}{M_{2}}N_{1}M\left(M-K\right)+\frac{J_{\phi }}{N_{1}}M_{2}M\left(M-K\right)\right)$
The iterative approach	$O\left(M^{3}+\left(\frac{J_{\theta }^{c}}{M_{2}}N_{1}+\frac{J_{\phi }^{c}}{N_{1}}M_{2}+J_{I}\left(M_{2}+N_{1}\right)\right)M\left(M-K\right)\right)$
